# Nursing students’ willingness to discuss hospice and palliative care with family and associated factors: A cross-sectional study

**DOI:** 10.1017/S1478951525100801

**Published:** 2025-10-01

**Authors:** Hui Li, Huaiting Gu, Tingting Xu, Qiushi Liu

**Affiliations:** 1 School of Nursing, Jining Medical University, Shandong, China; 2 School of Public Health, Jining Medical University, Shandong, China; 3 School of Nursing, Shandong First Medical University, Shandong, China; 4 International Nursing School, Hainan Medical University, Hainan, China

**Keywords:** Nursing students, hospice and palliative care, family, willingness, associated factors

## Abstract

**Objectives:**

In the cultural context of China, it holds profound significance for nursing students to engage in discussions about hospice and palliative care with their families. This study aimed to explore nursing students’ willingness to discuss hospice and palliative care with their families and the factors associated with it.

**Methods:**

Nursing students from three schools in three Chinese provinces (*n* = 1,234) completed questionnaires on general information, hospice and palliative care awareness, attitude toward death, and willingness to discuss hospice and palliative care with their families. This cross-sectional analysis utilized logistic regression to investigate the predictors of participants’ willingness to discuss hospice and palliative care with their families.

**Results:**

The mean hospice and palliative care knowledge score was 6.68, and 19.1% were willing to discuss the topic with their families. Factors associated with nursing students’ willingness to discuss hospice and palliative care with their families included region, whether their family members considered talking about death a taboo, whether a family member was severely ill and at risk of death, their knowledge of World Hospice and Palliative Care Day, hospice and palliative care knowledge score, and death avoidance attitude. Participants with higher hospice and palliative care knowledge scores were more willing to discuss the topic with their families, while a higher death avoidance score was associated with unwillingness.

**Significance of results:**

Nursing students significantly lack hospice and palliative care awareness, and their willingness to discuss the topic with their families needs improvement. Nursing schools should provide systematic and standardized hospice and palliative care education and communication skills training.

## Introduction

In traditional Chinese culture, “death” is perceived as an unlucky symbol, and the Confucian dictum of “one does not know life before knowing death” has long suppressed familial discussions on end-of-life issues, particularly when confronted with terminal illness. Despite family members occupying a central role in medical decision-making, studies indicate discrepancies in preferences and attitudes toward end-of-life treatments between individuals and their families (Ye et al. 2023; Zhu et al. 2023), placing immense pressure on family members during decision-making (Hu et al. 2024; Shen et al. 2023). The incidence of decision regret among patients’ relatives ranges from 27.0% to 52.7% (Zhang et al. 2024), with inadequate familial discussions on death being one of the primary reasons (Mori et al. 2017). Promoting open familial discussions on hospice and palliative care (HPC) is not only an ethical imperative to respect patients’ autonomy but also a crucial path to breaking intergenerational silence and enhancing the quality of death (Yamaguchi et al. 2017).

Public awareness of HPC is generally inadequate (Lin et al. 2022; Shen et al. 2023; Ye et al. 2023; Zhu et al. 2020), and acceptance levels vary. Families with higher educational attainment and social status are more likely to embrace advance care planning (Lin et al. 2022; Shen et al. 2023; Wang et al. 2024). Ordinary families, however, face cognitive barriers owing to cultural taboos and knowledge scarcity and even misinterpret HPC as “abandoning treatment,” especially in economically underdeveloped regions (Lin et al. 2022; Yang and Jia 2022). The need to reshape societal perceptions through community education, family meetings, and other multidimensional interventions is urgent (Tu and Mei 2024). Notably, healthy individuals are more receptive to advance care planning (Davies et al. 2023), providing a breakthrough for initiating familial dialogue earlier.

As the core force of the future health care system, nursing students are not only recipients and practitioners of HPC education but also cultural intermediaries connecting families and the medical system. Notably, the majority of Chinese nursing students come from ordinary families (Su et al. 2024), and their upbringing overlaps significantly with the group most resistant to HPC promotion. This background enables nursing students to understand communication barriers owing to familial cultural taboos while possessing the potential to break through traditional notions via professional education. Studies indicate that nursing students’ discussions on death with their families positively influence their knowledge of and attitudes toward HPC (Jiang et al. 2019; Shi et al. 2020). Such discussions not only facilitate death education within families and disseminate the HPC concept but also serve as crucial training grounds for communication skills, significantly enhancing nursing students’ clinical empathy and decision-guiding skills. More profoundly, leveraging family trust, nursing students can form a dissemination chain of “one person influencing one household, one household influencing the community,” thereby promoting the HPC concept in private as well as public domains.

The culture of filial piety exhibits contradictory tensions in this process (Lin and Guo 2024): while suppressing open discussions on death owing to ethical requirements of “serving parents with utmost care,” it also provides a cultural fulcrum for dialogue by encouraging optimal care. The advocacy of World Hospice and Palliative Care Day (WHPCD) offers an opportunity to break taboos and transform the core of filial piety culture, that is, “respect,” into a positive force driving familial dialogue.

Based on the knowledge, attitude, and practice theory (Bettinghaus 1986) and against the backdrop of WHPCD, we focus on the willingness of nursing students to discuss HPC with their families in the Chinese cultural context; identify the resistance to and motivation behind family dialogue; and provide a basis for optimizing the curriculum design of nursing schools, as well as for constructing an HPC promotion model that conforms to the cultural characteristics of Chinese society.

## Methods

### Design

We used a cross-sectional design to develop the survey, which was conducted online through a commonly used survey platform (https://www.wjx.cn/).

### Instruments

#### Demographic characteristics

Demographic characteristics included gender, age, school, grade, religion, region, whether it was taboo to talk about one’s death, whether family members considered talking about death taboo, whether one had experienced the death of a family member, and whether one had a family member who was seriously ill or facing the risk of death. Participants could answer either “yes” or “no.”

#### HPC awareness

This included WHPCD knowledge (e.g., “have you heard of WHPCD?” and “the time and theme of WHPCD this year”). Participants completed the Palliative Care Quiz for Nursing (PCQN), which assesses students’ HPC knowledge (Ross et al. 1996). It comprises 20 questions with the following answer choices: “true,” “false,” and “don’t know.” The higher the score, the better the knowledge. The internal consistency of the Chinese version was 0.758 (Zou 2007).

#### Attitude toward death

The Chinese version of the Death Attitude Profile–Revised was used to assess death attitude (Xie et al. 2021). The scale comprises 32 items across five dimensions: fear of death, escape death, natural acceptance, approach acceptance, and escape acceptance. The items are rated on a 5-point Likert scale ranging from 1 (“strongly disagree”) to 5 (“strongly agree”). Each dimension produces an average score, with a higher score indicating a greater death attitude tendency in that particular dimension. Cronbach’s α reliability coefficient was 0.87 in this study.

#### Willingness to discuss HPC with family members

This was assessed using the following “yes” or “no” questions: “Are you willing to discuss HPC with your family?” and “Do you think it is necessary to discuss HPC with your family?” Other questions included “Do you find it difficult to discuss HPC with your family?” and “What is the biggest difficulty?” Several answer options were provided for these questions.

### Sampling and recruitment

Students were recruited from three schools in three provinces (Beijing; Yueyang, Hunan Province; and Jining, Shandong Province) through convenience sampling. The researchers, who are teachers at the nursing schools, first described the study purpose to the students. They indicated that participation was voluntary, confidential, and anonymous. The researchers explained to the students that the questionnaire requested no identifiable personal information and that there would be no negative consequences for not participating or withdrawing. Interested participants could scan a QR code for access to the online survey link.

### Inclusion and exclusion criteria

The inclusion criteria for the participants were (a) being full-time students registered at the school, (b) majoring in nursing, and (c) providing written informed consent. The exclusion criteria were (a) being absent from class or applying for leave during the investigation and (b) a clinical diagnosis of mental disorders or serious psychological problems.

### Data analysis

SPSS version 23.0 (IBM Corp., Armonk, NY, USA) was used for data analysis. Descriptive statistics were employed to describe participant characteristics, PCQN scores, attitude toward death, and willingness to discuss HPC with family. A chi-squared test was used to analyze the association between participant demographic characteristics and their willingness to discuss HPC with their families. Further, a *t*-test was employed to analyze the associations of HPC knowledge (PCQN score) and attitude toward death with willingness to discuss HPC with their families. A logistic regression analysis was utilized to identify potential predictors of participants’ willingness to discuss HPC with their families. The *P*-value of the Hosmer–Lemeshow test was greater than 0.05, indicating that the model was a good fit.

### Ethical considerations

Each participant completed an informed consent sheet on the front page of the electronic questionnaire. This study’s protocol was approved by the Institutional Review Board of the authors’ affiliate university. To ensure there were no duplicate participants, we limited the number of times a participant could open the link using a mobile device to one. Data were issued and recalled on 12 October 2024 (WHPCD). The questionnaire took approximately 10 minutes to complete.

## Results

### Participants

Questionnaires were shared with 1,500 students, and 1,234 valid questionnaires were returned (effective recovery rate 82.3%). Participants were aged 17 to 29 (19.29 [±1.332]) years. Most were from higher vocational colleges (70.8%) and rural areas (74.1%). Further, the majority had no religious beliefs (88.7%). See Table 1 for details.

**Table 1.** Univariate analysis of participants’ willingness to discuss HPC with families and their demographic characteristics (*N* = 1,234)

Demographic characteristic	Response	*n* (%)	Unwilling, *n* (%)	Willing, *n* (%)	χ^2^	*P*-value
Gender	Man	108 (8.8)	84 (77.8)	24 (22.2)	0.734	0.392
	Woman	1,126 (91.2)	914 (81.2)	212 (18.8)		
Grade	Freshman	284 (23.0)	221 (77.8)	63 (22.2)	4.589	0.205
	Sophomore	760 (61.6)	620 (81.6)	140 (18.4)		
	Junior	115 (9.3)	99 (86.1)	16 (13.9)		
	Senior	75 (6.1)	58 (77.3)	17 (22.7)		
School	Undergraduate school	360 (29.2)	284 (78.9)	76 (21.1)	1.297	0.255
	Higher vocational college	874 (70.8)	714 (81.7)	160 (18.3)		
Region	Rural areas	914 (74.1)	756 (82.7)	158 (17.3)	7.699	0.006
	City/town	320 (25.9)	242 (75.6)	78 (24.4)		
Religion	Yes	139 (11.3)	116 (83.5)	23 (16.5)	0.673	0.412
	No	1095 (88.7)	882 (80.5)	213 (19.5)		
Is it taboo to talk about one’s death?	No	1,078 (87.4)	850 (78.8)	228 (21.2)	22.618	0.000
	Yes	156 (12.6)	148 (94.9)	8 (5.1)		
Do your family members consider talking about death taboo?	No	660 (53.5)	465 (70.5)	195 (29.5)	99.615	0.000
	Yes	574 (46.5)	533 (92.9)	41 (7.1)		
Have you lost any family members?	Yes	856 (69.4)	693 (81.0)	163 (19.0)	0.012	0.911
	No	378 (30.6)	305 (80.7)	73 (19.3)		
Is a family member seriously ill and at risk of death?	Yes	240 (19.4)	181 (75.4)	59 (24.6)	5.740	0.017
	No	994 (80.6)	817 (82.2)	177 (17.8)		
Have you heard of WHPCD?	Yes	441 (35.7)	342 (77.6)	99 (22.4)	4.903	0.027
	No	793 (64.3)	656 (82.7)	137 (17.3)		
Did you know the specific date of WHPCD before this survey?	Yes	298 (24.1)	233 (78.2)	65 (21.8)	1.834	0.176
	No	936 (75.9)	765 (81.7)	171 (18.3)		
Do you know the theme of WHPCD this year?	Yes	332 (26.9)	257 (77.4)	75 (22.6)	3.527	0.060
	No	902 (73.1)	741 (82.2)	161 (17.8)		
Have you received HPC education?	No	1,036 (84.0)	847 (81.8)	189 (18.2)	3.244	0.072
	Yes	198 (16.0)	151 (76.3)	47 (23.7)		
Do you need to acquire HPC knowledge?	No	521 (42.2)	437 (83.9)	84 (16.1)	5.254	0.022
	Yes	713 (57.8)	561 (78.7)	152 (21.3)		

*Note*: HPC, hospice and palliative care; WHPCD, World Hospice and Palliative Care Day.

### HPC awareness

Participants’ average PCQN score was 6.68, with the highest average score obtained for the natural acceptance dimension of death attitude (Table 2). A total of 441 participants (35.7%) had heard of WHPCD, 298 students (24.1%) knew the specific date, and 332 students (26.9%) knew the theme for the year of the study. However, 1,036 (84.0%) had not received HPC education, and 713 (57.8%) thought they needed to obtain HPC-related knowledge (Table 1).

**Table 2.** Analysis of the association of PCQN score and attitude toward death with participants’ willingness to discuss HPC with family members

Item	*N* = 1,234	Unwilling (*n* = 998) Mean (standard deviation)	Willing (*n* = 236) Mean (standard deviation)	*t*	*P*-value
PCQN	6.68 (3.93)	6.44 (3.95)	7.67 (3.69)	−4.518	.000
Fear of death subscale	3.06 (0.69)	3.10 (0.67)	2.91 (0.75)	3.522	.000
Death avoidance subscale	3.10 (0.73)	3.14 (0.70)	2.90 (0.80)	4.317	.000
Natural acceptance subscale	3.59 (0.66)	3.58 (0.64)	3.63 (0.75)	−0.959	.338
Approach acceptance subscale	2.95 (0.65)	2.95 (0.62)	2.95 (0.77)	−0.071	.944
Escape acceptance subscale	2.87 (0.76)	2.87 (0.74)	2.88 (0.84)	−0.154	.878

*Note*: PCQN, Palliative Care Quiz for Nursing; HPC, hospice and palliative care.

### Willingness to discuss HPC with family

Of the total participants, 236 (19.1%) were willing to discuss HPC with their families. Further, 701 (56.8%) thought it was necessary to discuss HPC with their families. In total, 1,107 participants (89.7%) felt that they had difficulties discussing HPC with their families. The top four difficulties were “taboo surrounding death in traditional culture” (716, 64.7%), “lack of life and death-related education” (655, 59.2%), “lack of awareness about the benefits of HPC knowledge” (653, 59%), and “ignorance of HPC” (541, 48.9%).

### Factors associated with willingness to discuss HPC with family

The univariate analysis showed that the following factors were significantly correlated with nursing students’ willingness to discuss HPC with their families: whether it was taboo to talk about death, whether their family members considered talking about death a taboo, whether a family member was severely ill and at risk of death, whether one needs to acquire HPC knowledge, WHPCD knowledge, PCQN score, fear of death, and death avoidance attitude (Tables 1 and 2).

The logistic regression results showed that region (odds ratio (OR) = 1.48, 95% confidence interval (CI) [1.06, 2.06]), whether their family members considered talking about death a taboo (OR = 5.02, 95% CI [3.43, 7.34]), whether a family member was severely ill and at risk of death (OR = 1.47, 95% CI [1.02, 2.12]), WHPCD knowledge (OR = 1.39, 95% CI [1.01, 1.90]), PCQN score (OR = 1.10, 95% CI [1.05, 1.14]), and death avoidance attitude (OR = 0.67, 95% CI [0.49, 0.93]) were associated with nursing students’ willingness to discuss HPC with their families (Table 3).

**Table 3.** Results of the logistic regression analysis on the factors associated with nursing students’ willingness to discuss HPC with their families (0 = no; 1 = yes; *N* = 1,234)

						95% CI for OR	
	B	SE	Wals	*P*-value	OR	Lower	Upper
Region	0.390	0.170	5.282	.022	1.48	1.06	2.06
Rural areas = 0, City/town = 1							
Is it taboo to talk about one’s death?	0.632	0.398	2.514	.113	1.88	0.86	4.11
Yes = 0, No = 1							
Do your family members consider talking about death taboo?	1.613	.194	68.959	.000	5.02	3.43	7.34
Yes = 0, No = 1							
Is a family member seriously ill and at risk of death?	0.382	.187	4.164	.041	1.47	1.02	2.12
No = 0, Yes = 1							
Have you heard of WHPCD?	0.326	0.162	4.059	.044	1.39	1.01	1.90
No = 0, Yes = 1							
Do you need to acquire HPC knowledge?	−0.286	0.165	3.029	.082	0.75	0.54	1.04
Yes = 0, No = 1							
PCQN	0.092	0.022	17.965	.000	1.10	1.05	1.14
Fear of death subscale	−0.048	0.175	0.074	.785	0.95	0.68	1.34
Death avoidance subscale	−.394	0.164	5.732	.017	0.67	0.49	.93
Constant	−2.613	0.574	20.712	.000	0.073		

*Note*: PCQN, Palliative Care Quiz for Nursing; HPC, hospice and palliative care; WHPCD, World Hospice and Palliative Care Day; CI, confidence interval; SE, standard error; OR, odds ratio. * *P* < .05.

## Discussion

To the best of our knowledge, this is the first study on nursing students’ willingness to discuss HPC with their families. Specifically, we investigated the willingness of 1,234 nursing students to discuss HPC with their families and the factors associated with their motivation to do so. Only 19.1% were willing to discuss HPC with their families, although 56.8% thought it was necessary. This indicated that the nursing students had a low willingness but a relatively positive attitude regarding discussing HPC with their families. This was lower than nurses’ willingness to discuss HPC with dying patients (61.9%) and their families (72.2%) (Lin et al. 2021). This may be related to nursing students’ lack of HPC discussion experience. All in all, 89.7% reported difficulties in discussing HPC with their families. The main barriers to HPC discussions were the taboo surrounding death in traditional culture and a lack of related education.

We demonstrated that 64.3% of the nursing students surveyed had not heard of WHPCD, and the PCQN score was 6.68, lower than in other countries (Dimoula et al. 2019; Gelegjamts et al. 2020). This indicates a low awareness of HPC among nursing students. HPC is yet to develop into an independent discipline in mainland China, with only a few specialized HPC courses provided in nursing schools. Related concepts are scarce in other courses, and neither a systematic theoretical knowledge system nor a mature and standardized education model has been created (Ling et al. 2020). None of the three schools in this study offered independent courses on HPC, and 84.0% of the nursing students had not received meaningful education on HPC. Without relevant education and training, nursing students face limitations regarding their HPC awareness, which may undermine their confidence and willingness to discuss HPC with their families.

Our results suggest that urban nursing students may be more willing to discuss HPC with their families than rural nursing students. This is inconsistent with Gelegjamts et al.’s (2020) findings, likely owing to China’s culture of avoiding death, which is especially prominent in the countryside. Nursing students whose family members do not consider talking about death a taboo may be more willing to discuss HPC. For such family members, HPC discussions may take place in a more natural, honest, in-depth, open, and calm manner. Nursing students will not feel nervous and pressured, meaning they might be more likely to talk about HPC. Those with a severely ill family member at risk of death may see their pain, recognize their needs, and realize the importance of HPC. Therefore, they may be more willing to discuss HPC with their families. Further, the experience of caring for a dying family member leads to a more positive HPC attitude among nursing students (Jiang et al. 2019).

Nursing students who knew about WHPCD had a better understanding of the purpose and value of HPC, which may enhance their willingness to discuss HPC with their families. WHPCD exposes students to foundational HPC concepts that their non-aware peers may not have been exposed to. Annually, WHPCD initiatives – such as promotional activities in hospitals, communities, and academic institutions – provide critical engagement opportunities. In addition to offline publicity, we recommend strengthening its online publicity, using the Internet and WeChat public accounts to promote broader engagement with WHPCD among nursing students.

Nursing students with a high PCQN score were more likely to discuss HPC with their families, which was consistent with previous studies (Leung and Wong 2021; Nedjat-Haiem et al. 2019). Moreover, HPC knowledge can increase students’ confidence in nursing practice (Leung and Wong 2021). Nursing students who have more knowledge about HPC may be more confident discussing HPC with their families; indeed, education and training can improve students’ HPC knowledge (Tamaki et al. 2019). Our results illustrated that 57.8% of nursing students thought they needed HPC knowledge, indicating that they had a positive attitude toward HPC education. Those students with death avoidance tendencies may be reluctant to discuss HPC with their families. Additionally, those who hold avoidance attitudes toward death may avoid the topic of death and be less willing to actively discuss it. In a previous study, students who avoided the topic of death had a more negative attitude toward HPC (Petrongolo and Toothaker 2021). Professional education helps individuals recognize death, regard death as a normal part of life, and overcome the fear of and desire to escape death (Gocmen Baykara et al. 2022).

Considering these results, it is necessary to strengthen and standardize HPC education for nursing students in mainland China. Many countries worldwide have identified HPC as a mandatory course in nursing education (Martins Pereira et al. 2021) and use a mix of educational strategies to improve students’ theoretical knowledge and skills (Carmack and Kemery 2018). Communication skills are one of the core competencies of HPC (Hökkä et al. 2024; Nilsson et al. 2022), and the training of communication skills in HPC for Chinese nursing students should be strengthened. Educators should also consider the cultural differences between the East and West and develop teaching programs suitable to both the culture and clinical practice in China.

In this study, no correlation was found between religion and nursing students’ willingness to discuss HPC with their families, which is inconsistent with other studies (Jiang et al. 2019; Shi et al. 2020). The reason may be that nursing students do not clearly understand religious beliefs, and their religious beliefs are based on half-understood concepts formed by family members’ influence.

### Strengths and limitations

Although we used a large sample and provided valuable information, there were several limitations. First was the use of a convenience sampling method with only three provinces participating, which may limit the generalizability of the findings. Second, this was a cross-sectional study, limiting causal inferences. Finally, the willingness scale is a potential limitation because its validity and reliability have not been established, and this might introduce some bias.

Based on the findings of this study, future projects can be designed and implemented to provide HPC education and communication skills training for nursing students, with their effectiveness assessed through randomized controlled trials.

## Conclusion

As the backbone of the future health care system, nursing students’ willingness and ability to discuss HPC with families directly impact the promotion and practice of HPC. We found that nursing students lack sufficient HPC knowledge and communication willingness, reflecting the profound influence of cultural taboos and educational deficiencies on their behavior. We also identified the factors relevant to nursing students’ willingness to discuss HPC with their families, among which HPC knowledge scores were positively correlated with discussion willingness, indicating that knowledge is an important driver of open dialogue between nursing students and their families. It is recommended that nursing schools systematically incorporate HPC courses, focusing on enhancing the students’ knowledge levels and communication skills, particularly sensitivity training regarding attitudes toward death and cultural taboos.

## Data statement

The raw dataset used to support the findings of the present study is available from the corresponding author upon request.
